# Environmental Enrichment Reverses Histone Methylation Changes in the Aged Hippocampus and Restores Age-Related Memory Deficits

**DOI:** 10.3390/biology4020298

**Published:** 2015-04-01

**Authors:** Sarah J. Morse, Anderson A. Butler, Robin L. Davis, Ian J. Soller, Farah D. Lubin

**Affiliations:** Department of Neurobiology, The Evelyn F. McKnight Brain Institute, University of Alabama at Birmingham, 1825 University Boulevard, Birmingham, AL 35294, USA; E-Mails: umbraphiles@gmail.com (S.J.M.); aabutler@uab.edu (A.A.B.); robinlynndavis@gmail.com (R.L.D.); ijsoller@uab.edu (I.J.S.)

**Keywords:** epigenetics, histone acetylation, gene expression, memory, chromatin remodeling, hippocampus, methyltransferases, demethylases

## Abstract

A decline in long-term memory (LTM) formation is a common feature of the normal aging process, which corresponds with abnormal expression of memory-related genes in the aged hippocampus. Epigenetic modulation of chromatin structure is required for proper transcriptional control of genes, such as the brain-derived neurotrophic factor (*Bdnf*) and *Zif268* in the hippocampus during the consolidation of new memories. Recently, the view has emerged that aberrant transcriptional regulation of memory-related genes may be reflective of an altered epigenetic landscape within the aged hippocampus, resulting in memory deficits with aging. Here, we found that baseline resting levels for tri-methylation of histone H3 at lysine 4 (H3K4me3) and acetylation of histone H3 at lysine 9 and 14 (H3K9,K14ac) were altered in the aged hippocampus as compared to levels in the hippocampus of young adult rats. Interestingly, object learning failed to increase activity-dependent H3K4me3 and di-methylation of histone H3 at lysine 9 (H3K9me2) levels in the hippocampus of aged adults as compared to young adults. Treatment with the LSD-1 histone demethylase inhibitor, t-PCP, increased baseline resting H3K4me3 and H3K9,K14ac levels in the young adult hippocampus, while young adult rats exhibited similar memory deficits as observed in aged rats. After environmental enrichment (EE), we found that object learning induced increases in H3K4me3 levels around the *Bdnf*, but not the *Zif268*, gene region in the aged hippocampus and rescued memory deficits in aged adults. Collectively, these results suggest that histone lysine methylation levels are abnormally regulated in the aged hippocampus and identify histone lysine methylation as a transcriptional mechanism by which EE may serve to restore memory formation with aging.

## 1. Introduction

Hippocampus-dependent long-term memory (LTM) deficits are well documented as part of the normal aging process in both humans and rodent animal models [1,2]. These age-related memory deficits occur without significant alterations in gross hippocampal morphology [3]. Instead, such memory deficits are reflective of a general dysregulation of memory-permissive genes within the aged hippocampus that are crucial to the process of LTM formation, including deviations in immediate-early genes like brain-derived neurotrophic factor (*Bdnf*) and *Zif268* (also known as *Egr1*) [4,5,6,7,8,9,10,11,12]. A growing literature suggests that epigenetic modulation of chromatin structure around gene regions is a transcriptional mechanism necessary for the formation and maintenance of LTM [10,12,13]. Therefore, alterations in the epigenetic landscape within the aged hippocampus may result in activity-dependent perturbations of gene transcription changes necessary for proper LTM formation [14,15,16,17].

Histone lysine methylation is a unique epigenetic transcriptional regulator whose function relies on the recruitment of proteins to regulate chromatin structure and subsequent cellular transcriptional activity (for review, see [18,19]). During the process of memory consolidation, tri-methylation of histone H3 at lysine 4 (H3K4me3) and di-methylation of histone H3 lysine 9 (H3K9me2) are involved in the upregulation or downregulation of genes, respectively, within the hippocampus and disruption of these epigenetic mechanisms produces memory deficits [12,20]. With respect to memory deficits in normal aging, the role of histone lysine methylation remains to be explored. Additionally, environmental enrichment (EE) reverses age-related cognitive decline [21,22,23,24,25], yet the effect of EE on histone lysine methylation changes in the aged hippocampus in response to learning is currently unknown. This prompted us to explore the possibility that EE interacts with histone lysine methylation transcriptional mechanisms to restore proper memory formation with aging.

## 2. Methods

***Animals:*** Young adult (3 months) and aged adult (19–22 months) male Fischer-344 rats (National Institute on Aging at Harlan) were used in these experiments. Animals were singly housed under light/dark 12 h/12 h and allowed access to food and water ad libitum. Animals were handled 3–5 min each and allowed to acclimate to laboratory conditions for 5 days prior to experiments. All procedures were performed in accordance with the Institutional Animal Care and Use Committee at the University of Alabama at Birmingham and national guidelines and policies.

***Environmental enrichment:*** Rats were transported to the laboratory 30 min before enrichment or handling. Aged rats were introduced pair-wise to one another in the empty enrichment cage for 5 min per session for 2 days to reduce stress and possible aggression with a maximum of 3 sessions per animal per day. All animals were subsequently exposed to EE for 1 h per day for 5 weeks. EE consisted of a large cage (76.2 cm × 76.2 cm × 25.4 cm) in which the animals were able to interact socially and explore tunnels, plastic blocks and balls, ladders, and other toys. To avoid behavioral habituation, additional new toys were introduced throughout the 5 week-period and objects were rearranged daily. Running wheels were specifically excluded to avoid neurogenesis effects in the dentate gyrus and the associated effects on gene expression arising from exercise-mediated alterations of synaptic plasticity [26,27,28]. Control animals remained in standard housing conditions and were transported alongside the EE animals followed by placement in a separate room where they received similar amounts of handling.

***Novel object recognition:*** In the novel object recognition (NOR) task rats were individually placed in a 31 cm × 31 cm box, which was covered with a white sheet and cleaned with 50% isopropanol before and after each trial. On day 1 of NOR, rats were exposed to the empty arena for 5 min for habituation to the environment to reduce stress during subsequent trial phases. After 24 h, animals were exposed to a 10 min training session with two identical sample objects. For biochemistry studies, one set of animals was sacrificed 1 h after participation in this training session for tissue harvesting, during the consolidation phase of memory formation in which de novo protein synthesis occurs. During the testing phase, animals’ memory performances were assessed via their ability to discriminate between a novel and familiar object [29]. This testing phase occurred 24 h after the training phase and lasted for a 5 min duration. Sample and novel objects were cleaned after each trial and positions were randomly exchanged throughout trials. All objects were weighted and adhered to the floor to avoid movement out of the scoring area. Trials were recorded using the Noldus Ethovision software, using the Phenotyper camera box (Noldus) placed directly above the arena. Trials were scored by a researcher blind to the animal’s identity. Contact with objects was defined as previously described [30]. Drug—Animals were intraperitoneally (IP) injected with either saline (0.9% NaCl, pH 7.4) or trans-2-Phenylcyclopropylamine hydrochloride (3 mg/kg, Sigma Chemical).

***Object Location:*** In the Object Location (OL) task rats were individually placed in a 31 cm × 31 cm box, which was covered with a white sheet and cleaned with 50% isopropanol before and after each trial. On day 1 of NOR, rats were exposed to the empty arena for 5 min for habituation to the environment to reduce stress during subsequent trial phases. After 24 h, animals were exposed to a 10 min training session with two identical sample objects in predetermined locations. All objects were weighted and adhered to the floor to avoid movement out of the scoring area. During the testing phase, animals’ memory performances were assessed via their ability to discriminate between objects in novel or familiar locations (NL or FL, respectively). This testing phase occurred 24 h after the training phase and lasted for a total of 5 min. Objects were cleaned after each trial and the object in the novel location was randomly exchanged throughout trials. Trials were as described above. Trials were scored by a researcher blind to the animal’s identity. Contact with objects was defined as previously described [30].

***Tissue collection:*** After decapitation, brains were removed and hemisected. One half of the brain was cut at the optic chiasm and 5 mm posterior to the brain, placed in cassettes with freezing medium on dry ice and flash-frozen at −80 °C. Slices were made using a LEICA cryostat cleaned with RNase inhibitor and UV-sterilized prior to use. Four 10 μm single-hemisphere slices were mounted onto PEN membrane glass slides (Applied Biosystems) for use in laser-capture microdissection. The remaining hemisphere was immersed in oxygenated (95%/5% O_2_/CO_2_) ice-cold cutting saline (containing the following (in mM): 110 sucrose, 60 NaCl, 3 KCl, 1.25 NaH_2_PO_4_, 28 NaHCO_3_, 0.5 CaCl_2_, 7 MgCl_2_, 5 glucose, 0.6 ascorbate) prior to removal of the whole hippocampus and subdissection under a dissection microscope for isolation of areas CA1, CA3, and dentate gyrus. These samples were frozen on dry ice and stored at −80 °C.

***Laser-capture microdissection:*** Hippocampal region CA1 pyramidal layer tissue samples were collected via laser-capture microdissection. Hippocampal slices were dried with 70% EtOH followed by rehydration with Milli-Q H2O (Millipore, Billerica, MA, USA). Next, tissues were stained with 1% cresyl violet staining solution (Sigma-Aldrich, St. Louis, MO, USA) with 5% SUPERase-IN RNase inhibitor (Ambion (now Life Technologies), Grand Island, NY, USA) followed by additional application of 1% cresyl violet solution without RNase inhibitor. Excess cresyl violet was washed with H_2_O followed by 70% EtOH, 30 s, 95% EtOH, 30 s, and 2 washes of 100% EtOH, 30 s. Xylene was added to tissues and allowed to dry prior to loading into the LCM machine. Laser settings used for microdissection were power: 75–85 mW and pulse: 1600–3000. Cells were captured onto CapSure HS LCM Caps (Arcturus (now Life Technologies), Grand Island, NY, USA), incubated for 30 min at 42 °C in extraction buffer from the AllPrep DNA/RNA mini kit (Qiagen, Venlo, Netherlands). Total RNA was extracted immediately after this incubation according to the manufacturer’s instructions. RNA was stored at −80 °C.

***Real-time RT-PCR:*** Total RNA extracted was quantified spectrophotometrically using a Thermo-Scientific Nano-Drop. mRNA was reverse transcribed using the iScript cDNA synthesis kit (Bio-Rad, Hercules, CA, USA) according to the manufacturer’s instructions. PCR amplifications were performed either in an iQ5 real-time PCR system (Bio-Rad), or in a CFX96 real-time PCR system (Bio-Rad), using Biorad iQ SYBR mastermix, SsoAdvanced SYBR mastermix according to manufacturer’s instructions.

***Histone extraction:*** Histone extractions were performed as previously described [12,20,31]. Briefly, all procedures were performed on ice with solutions chilled to 4 °C and all centrifugation steps were performed at 4 °C. Tissue from each hippocampal subfield was Dounce homogenized using no more than 6 strokes of a glass pestle (Kontes Glass) in ice-cold homogenization buffer containing the following (in mM): 250 sucrose, 50 Tris, pH 7.5, 25 KCL, 0.5 phenymethylsulfonyl fluoride, 1% protease inhibitor mixture (Sigma, St. Lous, MO, USA), and 0.9 Na+-butyrate. Tissue homogenates were centrifuged at 7700× *g* for 1 min. The pellet was resuspended in 0.5 mL of 0.4N H_2_SO_4_, incubated for 30 min, and centrifuged at 14,000× *g* for 10 min. Proteins were precipitated from the supernatant by the addition of 250 μL of 100% trichloroacetic acid containing 4 mg/mL deoxycholic acid (Na+ salt, Sigma) for 30 min. Histone proteins were collected by centrifugation at 14,000× *g* for 30 min and the resulting pellet was washed with 1 mL acidified acetone (0.1% HCl) followed by 1 mL acetone for 5 min each. Between washes, protein precipitates were collected by centrifugation at 14,000× *g* for 5 min. Finally, the acid purified histone proteins were resuspended in 10 mM Tris, pH 8 and stored at −80 °C until Western blotting.

***Western blotting:*** Protein concentrations were determined using a DC protein assay (Bio-Rad) and aliquots of samples were normalized to 0.2 μg/μL. Laemmli sample buffer (final concentration: 6.25 mM Tris, pH 6.8, 2% SDS, 10% glycerol, 1.25% 2-mercaptoethanol, 0.1% bromophenol blue) was added to samples prior to performing SDS-PAGE on a 12% acrylamide resolving gel with a 4% acrylamide stacking gel. Histone proteins were transferred to Immobilon-FL polyvinylidene difluoride (PVDF) membranes (Millipore) for immunobloting, during which PVDF membranes were incubated in primary antibodies for 1 h at room temperature or overnight at 4 °C followed by incubation in secondary antibodies for 1 h at room temperature. Immunostained proteins were detected via the Odyssey Infrared Imaging System (LI-COR). Primary antibodies were obtained from Millipore Biotechnology and diluted in 1:1 PBST:Odyssey Blocking Buffer (LI-COR) as follows: anti-H3K4me3 (1:500), anti-H3K9me2 (1:500), and anti-H3 (1:1000). In all cases, the primary antibody host was rabbit. The secondary antibodies were goat anti-rabbit IRDye 800CW and IRDye 700DX fluorescent antibodies (LI-COR) diluted 1:20,000 in 1:1 PBS:Odyssey Blocking Buffer (LI-COR).

***Chromatin immunoprecipitation:*** ChIP analysis was performed as previously described [12,20]. Briefly, area CA1 of the hippocampus was microdissected and placed in ice-cold PBS solution containing protease inhibitors (1 mM phenylmethylsulfonyl fluoride, 1 μg/mL protease inhibitor cocktail (Sigma) and phosphatase inhibitors (1 mM Na3VO4 and 10 mM NaF)). Tissue was incubated in 1% formaldehyde in PBS at 37 °C for 10 min prior to homogenization in SDS lysis buffer (50 mM Tris, pH 8.1, 10 mM EDTA, 1% SDS). Chromatin was sheared using a Branson Sonifier 250 at 1.5 power and constant duty cycle. Lysates were centrifuged to remove debris and then diluted 1:10 in ChIP dilution buffer (16.7 mM Tris, pH 8.1, 0.01% SDS, 1.1% Triton X-100, 167 mM NaCl, 1.2 mM EDTA). Immunoprecipitations were carried out at 4 °C overnight with the primary antibody (anti-H3K4me3) or no antibody (control). Immune complexes were collected with a protein A-agarose bead/salmon sperm slurry and then washed with low salt buffer (20 mM Tris, pH 8.0, 0.1% SDS, 1% Triton X-100, 2 mM EDTA, 150 mM NaCl), high salt buffer, (20 mM Tris, pH 8.1, 0.1% SDS, 1% Triton X-100, 500 mM NaCl, 1 mM EDTA), LiCl immune complex buffer (0.25 M LiCl, 10 mM Tris, pH 8.1, 1% deoxycholic acid, 1% IGEPAL-CA630, 500 mM NaCl, 2 mM EDTA), and TE buffer. 1× TE containing 1% SDS was used to extract immune complexes. Protein-DNA cross-links were reverted by heating at 65 °C overnight and proteins were digested by proteinase K (100 μg, 2 h at 37 °C). DNA was extracted by phenol/chlorophorm/isoamyl alcohol and ethanol-precipitated. Immunoprecipitated DNA was analyzed via quantitative real-time PCR using primers specific for 150–200 bp segments corresponding to promoters upstream of the rat Bdnf or Zif268 transcription start site.

## 3. Results

### 3.1. Baseline Resting Hippocampal Histone Lysine Methylation Levels in Young and Aged Adults

**Figure 1:**
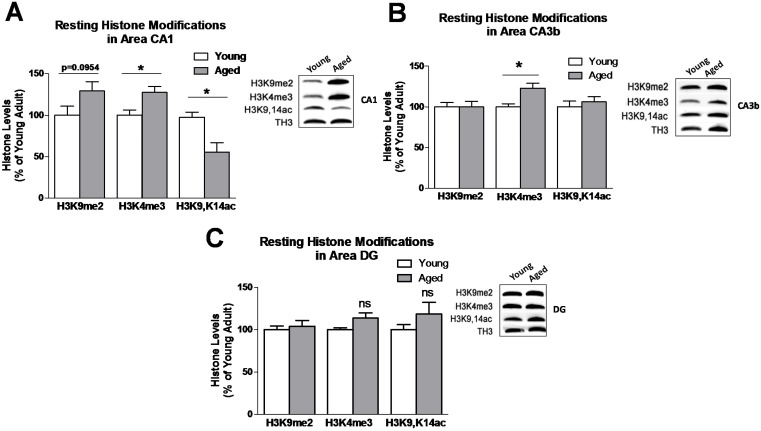
Resting histone modification levels in the aging hippocampus. Animals were sacrificed directly from their home cages and histone modification levels were assessed in aged adults (19–22 mo) compared to young adults (3 mo). In area CA1 (**A**), we observed significantly different resting levels of H3K4me3 and H3K9,K14ac between young and aged animals (young H3K9me2, *n* = 5; aged H3K9me2, *n* = 6; young H3K4me3, *n* = 9; aged H3K4me3, *n* = 10; young H3K9,K14ac, *n* = 6; aged H3K9,K14ac, *n* = 6); In region CA3b (**B**), we observed increased H3K4me3 (young H3K9me2, *n* = 6; aged H3K9me2, *n* = 6; young H3K4me3, *n* = 4; aged H3K4me3, *n* = 6; young H3K9,K14ac, *n* = 6; aged H3K9,K14ac, *n* = 6). In the dentate gyrus (DG) region (**C**), we observed no differences in resting state levels of examined histone modifications between age groups (young, *n* = 6; aged, *n* = 6). Histone levels are presented as a percentage of the young adult group. *Student’s t-test*; *p < 0.05 compared to young adults. Data are shown ± SEM.

To begin exploring the potential role of histone lysine methylation in age-associated LTM dysfunction, we first assessed baseline resting levels of two distinct H3 lysine methylation modifications (H3K9me2 and H3K4me3) within the hippocampus of aged adult rats as compared to young adult rats. Using Western blotting analysis, we observed a strong trend towards elevation, but non-significant increase in baseline resting H3K9me2 (Figure 1A; t_(9)_ = 1.863, *p* = 0.0954) and a significant increase in H3K4me3 levels (Figure 1A; t_(17)_ = 2.881, *p* = 0.0104) in area CA1 of the hippocampus from aged adults compared to young adults. As previously described [16,18], we found that baseline resting H3K9,K14ac levels were markedly reduced in area CA1 of aged adult rats (Figure 1A; t_(9)_ = 3.012, *p* < 0.05). In area CA3b of the hippocampus, baseline resting H3K4me3 levels were significantly elevated with aging compared to young adults (Figure 1B; t_(8)_ = 2.843, *p* < 0.05), while baseline resting H3K4me3 levels in the dentate gyrus (DG) region remained unchanged (Figure 1C). Interestingly, no age-related alterations were observed in H3K9me2 or H3K9,K14ac levels in CA3b or DG, suggesting that age-related widespread disruption of histone-mediated gene transcription primarily occurred in area CA1 of the hippocampus. Furthermore, these results indicate that aberrant histone methylation levels, specifically H3K4me3, may contribute to transcriptional dysregulation in the aged hippocampus.

### 3.2. Learning induced Histone Lysine Methylation and Gene Expression Changes in the Young and Aged Hippocampus

**Figure 2:**
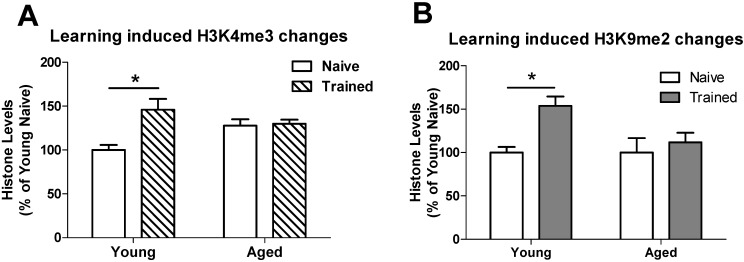
Learning-induced histone H3 methylation levels are altered in the aged hippocampus. Young and aged adults were sacrificed from their home cages (naïve) or at 1 h after training in NOR paradigm (Trained). Histone modification levels were detected by Western blotting and quantified by optical densitometry. H3K4me3 and H3K9me2 levels were assessed in area CA1 of the hippocampus. (**A**) Significant increases in H3K4me3 were detected in young-trained adults relative to young-naïve adults. A significant interaction between aging and learning (training) was detected, and learning-dependent increases in H3K9me2 levels seen in young adults were disrupted in aged adults; (**B**) Significant increases in H3K9me2 levels were detected in young-trained adults relative to young-naïve adults. No significant interaction was detected between aging and training; however, learning (training) induced increases in H3K9me2 observed in young animals were disrupted in aged animals. Results are presented as a percentage of young adult naïve. *Group sizes:* young naïve, *n* = 3; young trained, *n* = 5; Aged naïve, *n* = 4, Aged trained, *n* = 6. *Two-Way ANOVA*; *p < 0.05. Data are shown ± SEM.

We next determined whether learning triggers histone lysine methylation changes in the hippocampus of aged adult rats as compared to young adult rats. Because we found that posttranslational modifications of histone proteins were primarily dysregulated in area CA1 of aged adult rats, we focused our remaining experiments in this region of the hippocampus. Using Western blotting analysis, we observed significant elevation in H3K4me3 (Figure 2A) and H3K9me2 (Figure 2B) levels in area CA1 of the hippocampus from young adult rats, but not aged rats at one hour after object learning.

**Figure 3:**
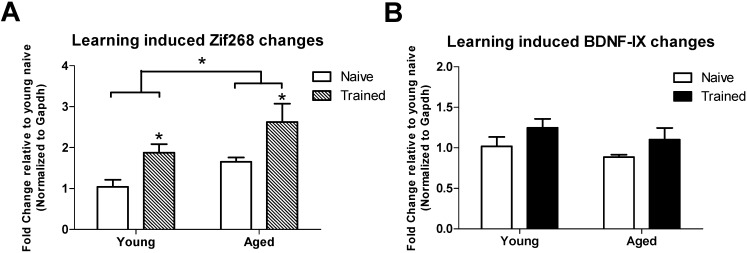
Learning induced changes in memory related gene expression in area CA1 of aged adults. Young and aged adults were sacrificed from their home cages (Naïve) or 1 h after training in NOR (Trained). H3K4me3 and H3K9me2 were assessed in hippocampal regions CA1. (**A**) *Zif268* mRNA levels are significantly increased after NOR training; (**B**) *Bdnf* mRNA levels were not significantly altered with training. Results are presented relative to young-naïve adults. *Group sizes*: young naïve, *n* = 4; young trained, *n* = 6; aged naïve, *n* = 4; aged trained, *n* = 6. *Two-Way ANOVA*; *p < 0.05. Data are shown ± SEM.

Next we investigated whether expression changes in the memory-related genes, *Zif268* (also known as *Egr1*) and *Bdnf exon IX* (a coding exon shared between all rat *Bdnf* transcript variants) correlated with histone lysine methylation changes in the hippocampus of aged adult rats as compared to young adult rats. We found that *Zif268* mRNA levels were significantly increased in area CA1 of the hippocampus from aged adult rats as compared to young adult rats (Figure 3A) and were observed to further increase following training in the novel object recognition (NOR) memory task (Figure 3A). We observed no significant changes in *Bdnf exon IX* mRNA levels (Figure 3B).

### 3.3. Inhibition of the LSD1 Histone Demethylase Mimics Age-Related Histone Lysine Methylation Changes and Memory Impairments in Young Adults

Based on the finding that baseline resting histone lysine methylation levels were significantly altered in the hippocampus of aged adults compared to young adults, we hypothesized that manipulating histone lysine methylation levels in young adults may produce similar effects on memory formation observed in aged adults. Therefore, we next sought to determine the effect of manipulating histone lysine methylation levels in young adult rats using the Lysine Specific Demethylase 1A (LSD1) inhibitor t-PCP [32]. We measured the effect of t-PCP on both baseline resting and behaviorally-induced H3K9me2, H3K4me3, and H3K9,14ac levels in the hippocampus of young adult rats.

**Figure 4:**
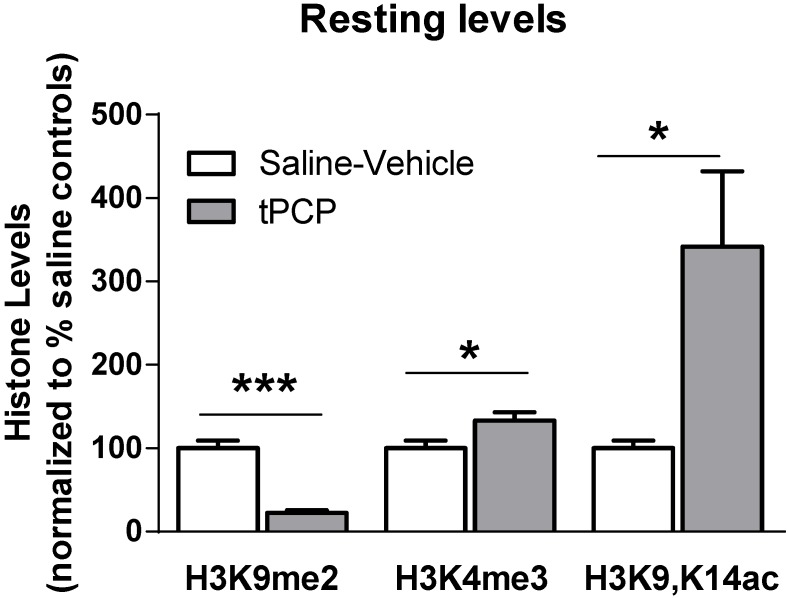
LSD1 inhibition alters baseline resting histone modification levels in area CA1 of young adults. Animals were given IP injections of 3 mg/kg t-PCP or saline-vehicle and the effect of LSD1 inhibition was assessed on H3K9me2, H3K4me3, and H3K9,K14ac levels in area CA1. Histone H3 modifications were normalized to total histone H3 protein levels. Histone modification levels are presented relative to the percentage of saline-vehicle controls. *Group sizes*: saline H3K9me2, *n* = 4; tPCP H3K9me2, *n* = 4; saline H3K4me3, *n* = 4; saline H3K9,K14ac, *n* = 4, tPCP H3K9,K14ac, *n* = 3. *Student’s t-test*; *p < 0.05, error bars represent the SEM.

First, we assessed baseline resting histone modification levels in area CA1 from saline-vehicle or t-PCP-treated animals not exposed to the NOR training arena or homecaged animals (Naïve). Western blotting analysis revealed that t-PCP treatment significantly increased baseline resting H3K4me3 and H3K9,K14ac levels in area CA1 from naïve adults, confirming that LSD1 inhibition successfully elevated histone methylation levels while simultaneously increasing histone acetylation levels in the hippocampus (Figure 4). Conversely, resting H3K9me2 levels were significantly reduced in area CA1 from t-PCP-treated adults compared to vehicle-treated controls (Figure 4). Together, these results may indicate a global shift towards more transcriptionally active chromatin (H3K4me3 and H3K9,K14ac) in area CA1 of t-PCP treated animals.

**Figure 5:**
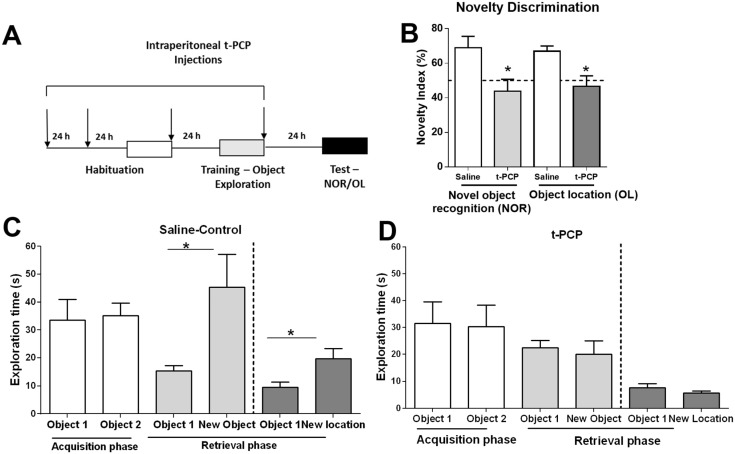
LSD1 inhibition impairs memory formation in young adults. (**A**) Diagram of the experimental design for the novel object recognition (NOR) and object location (OL) learning paradigms. Animals were IP injected with t-PCP or saline-vehicle as indicated; (**B**–**D**) LSD1 inhibition impaired performance in NOR and OL learning tasks; (**B**) Discrimination index [calculated as NS/(FS+NS)*100; FS = familiar stimulus; NS = novel stimulus], with an index ≥50% indicating levels of memory retention. Novelty exploration data for saline-vehicle controls (**C**) and t-PCP-treated animals (**D**) are presented as the total duration, in seconds, of exploration per object. During the training or acquisition phase, objects 1 and 2 represented two identical sample objects. During the testing or retrieval phase, object 1 represented the familiar object or location, and the new object or location are represented as such. *Group sizes NOR:* Saline, *n* = 6; tPCP, *n* = 4; *OL:* Saline, n = 5; tPCP, *n* = 5. *Student’s t-test*; ns = not significant, *p < 0.05, Error bars represent the SEM.

We next determined the effects of LSD1 inhibition with t-PCP on the formation of hippocampus-dependent memory. A schematic of our experimental design is outlined in Figure 5A. We assessed the impact of LSD1 blockade on LTM formation using two hippocampus-dependent novelty discrimination memory tasks: NOR and object location (OL). The discrimination index percentage (Figure 5B) and the time spent exploring each object was recorded (Figure 5C,D). We found that LSD1 inhibition significantly blocked memory formation in both the NOR and OL (Figure 5C,D) memory tasks relative to saline-vehicle treated controls. These results suggest that disruption of histone lysine methylation levels is sufficient to impair the formation of hippocampus-dependent memory in young adults.

### 3.4. Environmental Enrichment Alters Age-Related H3K4me3 Levels at Gene Regions during Memory Formation

Having established that alterations in H3K4me3 methylation were associated with age-related memory deficits and that mimicry of these age-related changes impaired LTM formation in young adults, we next explored the possibility of restoring appropriate H3K4me3 levels and rescuing memory deficits in aged animals through EE behavioral therapy. We first confirmed that age-associated memory impairments could be restored by a modified EE. The modified EE protocol used in the following experiments lacked the characteristic exercise components (*i.e.*, running wheel), as motor stimulation induces neurogenesis, which alone can result in gene expression changes, such as *Bdnf* within the DG region of the hippocampus [33,34,35].

**Figure 6:**
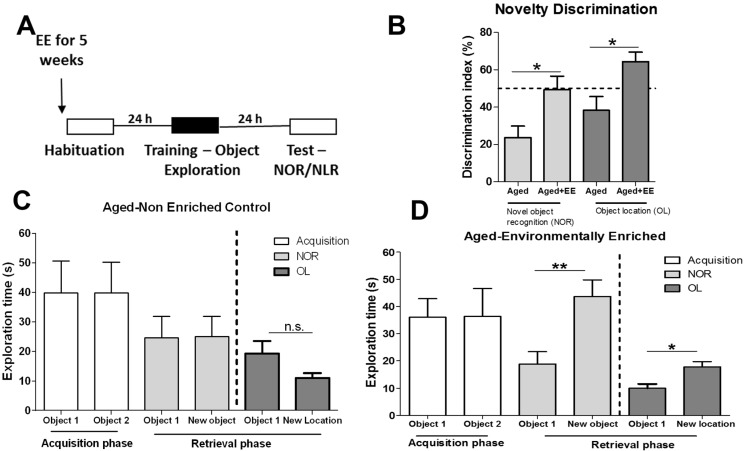
Environmental enrichment restores memory formation in aged adults. (**A**) Aged adults experience a 5-week EE protocol prior to behavioral experiments. Age-matched non-enriched controls received similar handling procedures; (**B**) A Novelty discrimination index indicate that memory impairment in aged adults was rescued with EE. Object exploration during training and testing phases (NOR and OL) for non-enriched aged controls (**C**) and aged-enriched adults (**D**) are presented as the total duration, in seconds, of exploration. Objects 1 and 2 represent the sample objects during acquisition. During the retrieval phase, object 1 represents the familiar object or familiar object location. *Group sizes NOR:* Aged, *n* = 5; Aged + EE, *n* = 6; *OL:* Aged, *n* = 4; Aged + EE, *n* = 5. *Student’s t-test*; *p < 0.05 between indicated groups. *p < 0.05, **p < 0.01 between indicated groups. Error bars represent SEM.

Aged adults were exposed to the modified EE protocol consisting of a variety of toys and social interaction for 1 h each day for a period of 5 weeks prior to exposure to the NOR and OL memory tasks (Figure 6A). Analysis of the discrimination index revealed that aged controls that did not receive EE demonstrated no preference for the novel object or novel location, which is characteristic of memory impairments (Figure 6B). Aged adults exposed to the EE showed a significant improvement over non-enriched controls in both NOR and OL memory tasks (Figure 6C,D).

No exploratory preference was detected during the acquisition phase and both non-enriched aged animals exhibited sufficient object interaction during the acquisition and retrieval phases thus ruling out the possibility that poor performance by the aged control adults was due to exploratory preferences or insufficient exploration during training (Figure 6C). EE aged adults exhibited a significant preference for the NO/NL during the retrieval phase (Figure 6D; t_(10)_ = 3.283, *p* < 0.01), confirming that EE restored LTM in aged adults. Furthermore, analysis of object interaction times for the aged-enriched group ensured that enhanced LTM formation was not due to increased motor activity during the 5-week EE protocol (Figure 6D). These results confirm that an exercise-free EE protocol improves LTM formation in aged adults.

**Figure 7:**
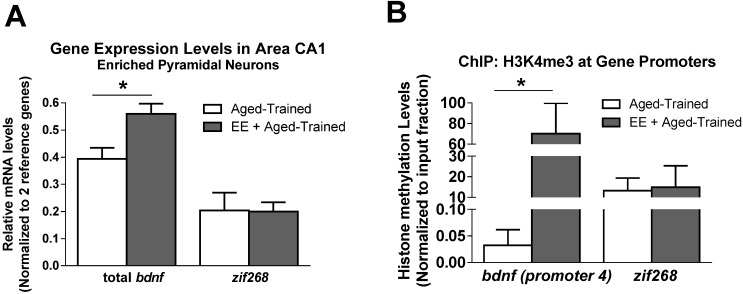
Environmental enrichment elevates H3K4me3 methylation levels at the *Bdnf* gene in the aged hippocampus. (**A**) Object Learning increases *Bdnf* mRNA level in the hippocampus of EE aged adults (*n* = 4) relative to non-enriched aged adults (*n* = 5); (**B**) ChIP analysis revealed EE dependent increases in H3K4me3 levels at the *Bdnf* promoter 4 during memory formation; *Group sizes for* (**B**): Aged-Trained, *n* = 4; EE + Aged-trained, *n* = 3. *Student’s t-test*; *p < 0.05 compared to non-enriched aged-trained adults. Data are shown ± SEM.

We next assessed the effect of EE on expression of the memory permissive genes *Bdnf* and *Zif268* in laser capture microdissection (LCM)-captured pyramidal neurons from area CA1. Prior to object learning aged animals were divided into two separate groups, one group experienced the 5-week EE protocol and the other group served as non-enriched aged controls. At 1 h after object training, we found that EE significantly increased *Bdnf*, but not *Zif268*, mRNA levels in LCM-captured pyramidal neurons from area CA1 during memory formation (Figure 7A; t_(7)_ = 2.923, *p* < 0.05).

Chromatin immunoprecipitation (ChIP) analysis revealed that H3K4me3 levels significantly increased at the *Bdnf exon 4* promoter (Figure 7B). We did not observe any significant activity dependent changes in H3K4me3 levels at the *Zif268* promoter region (Figure 7B), which is in agreement with our finding that EE did not alter activity dependent *Zif268* mRNA levels (Figure 7A). Together, these results strongly support histone lysine methylation mechanisms as molecular targets in the restoration of age-related memory by EE.

## 4. Discussion and Conclusions

Post-translational modifications of histone proteins, including histone phosphorylation, acetylation and methylation, have emerged as crucial regulators of transcriptional activity during LTM formation (reviewed in [18,36]). Interestingly, histone lysine methylation marks have been shown to cause downstream effects on mechanisms via recruitment of specific chromatin-modifying enzymes (Reviewed in [18,19,36,37,38]). Such studies suggest a critical role for histone lysine methylation in the development of age-associated cognitive deficits relative to histone acetylation; however, the role of hippocampal histone lysine methylation changes in the context of age-related cognitive decline had not been previously explored.

In the present study, we investigated the role of hippocampal histone lysine methylation levels changes in age-associated LTM impairments and made several important findings. We found that advanced age corresponds strongly with elevated resting H3K4me3 levels in multiple regions of the hippocampus. Additionally, we found that age-related memory impairment was strongly associated with alterations in histone lysine methylation levels in the aged hippocampus. Inhibition of the LSD1 histone demethylase in young adults resulted in increased H3K4me3 and H3K9,K14ac levels and decreased H3K9me2 levels in the hippocampus. We further observed that manipulating histone lysine methylation levels via inhibition of the LSD1 histone demethylase in young adults reproduced the age-associated increases in baseline resting H3K4me3 levels in area CA1 of the hippocampus, and similarly impaired performances in multiple hippocampus-dependent memory tasks. While we cannot discount the possibility of off-target effects resulting from systemic t-PCP treatment, previous work has demonstrated the ability of t-PCP and derived LSD1 inhibitors to be brain penetrant and impact hippocampus-dependent memory formation [26]. Given that LSD1 can target H3K9me2 for demethylation [39], one would expect increases in H3K9me2 levels with LSD1 inhibition; however, we observed significant decreases in H3K9me2. These results suggest a global shift towards more transcriptionally active chromatin, or alternatively a loss of gene silencing. Intriguingly, a similar effect is known to occur during the aging process; loss of heterochromatin [40]. Collectively, our results and these findings are suggestive of a shift from a transcriptionally repressed to a transcriptionally active epigenetic landscape with aging, which is consistent with observed changes in histone acetylation, yet produced memory impairments in young adults.

Next, we examined the effects of EE on histone methylation levels in the aged hippocampus and LTM formation impairments with age. We demonstrated that EE improves performance in hippocampus-dependent memory tasks and found that EE reversed learning-induced *Bdnf* gene expression concomitant with changes in H3K4me3 levels at the *Bdnf exon IV* promoter in the aged hippocampus. This suggests that histone lysine methylation changes in the aged-hippocampus are reversed with EE and associated with rescue of age-related memory impairments with EE therapy. Interestingly, previous studies have demonstrated that baseline histone lysine methylation marks including the repressive H3K9me3 and H3K27me3 marks are decreased at the *Bdnf* gene locus after three to four weeks EE in a mouse model [41]. While not directly comparable due to differences in study design, our present results add to these prior findings by demonstrating that learning induced differences in histone methylation in the aged hippocampus, which is in strong correlation with normalization of learning induced BDNF gene expression after EE. Together, these results suggest that EE regulates *Bdnf* expression through both baseline and learning induced changes in histone lysine methylation. Given the complex nature of the EE protocol, and its significant impact on animal health, it is quite likely that other potentially significant genes are also regulated. While our data suggest that these critical memory genes may be involved, recent studies suggest that histone lysine methylation pathways *themselves* are in fact a principal contributor to cognitive disorders [42].

In conclusion, we observed increased baseline resting histone lysine methylation levels (H3K4me3, H3K9me2) in the aged hippocampus. We found that manipulating baseline resting histone lysine methylation levels in area CA1 of young adults with previously intact memory led to the dysregulation of both histone methylation and acetylation levels in the hippocampus of young adults during memory formation, which resulted in memory impairments. EE reversed age-associated memory impairments and increased *Bdnf* transcription in association with increases in H3K4me3 levels at *Bdnf* promoter 4 in the aged hippocampus in response to object learning. These findings provide insights into histone lysine methylation-mediated transcriptional changes in the aged hippocampus, and implicate histone lysine methylation as a newly identified molecular mechanism affected by EE, involved in the restorative effect of EE on age-related memory deficits. Future studies should focus on the interrogation of EE-induced histone methyltransferases and histone demethylase activity to better identify potential therapeutic targets for the treatment of age-associated memory deficits.
